# Different responses of plant N and P resorption to overgrazing in three dominant species in a typical steppe of Inner Mongolia, China

**DOI:** 10.7717/peerj.9915

**Published:** 2020-09-18

**Authors:** Zhen Wang, Saheed Olaide Jimoh, Xiliang Li, Baoming Ji, Paul C. Struik, Shixian Sun, Ji Lei, Yong Ding, Yong Zhang

**Affiliations:** 1 Institute of Grassland Research, Chinese Academy of Agricultural Sciences, Hohhot, Inner Mongolia, China; 2 Sustainable Environment Food and Agriculture Initiative (SEFAAI), Lagos, Nigeria; 3 College of Forestry, Beijing Forestry University, Beijing, China; 4 Department of Plant Sciences, Wageningen University, Wageningen, Netherlands

**Keywords:** Livestock, Nutrient cycling, Leaf N concentration, Leaf P concentration, Nutrient resorption efficiency

## Abstract

Nutrient resorption from senesced leaves is an important mechanism for nutrient conservation in plants. However, little is known about the effect of grazing on plant nutrient resorption from senesced leaves, especially in semiarid ecosystems. Here, we evaluated the effects of grazing on N and P resorption in the three most dominant grass species in a typical steppe in northern China. We identified the key pathways of grazing-induced effects on N and P resorption efficiency. Grazing increased N and P concentrations in the green leaves of *Leymus chinensis* and *Stipa grandis* but not in *Cleistogenes squarossa*. Both *L. chinensis* and *S. grandis* exhibited an increasing trend of leaf N resorption, whereas *C. squarrosa* recorded a decline in both leaf N and P resorption efficiency under grazing. Structural equation models showed that grazing is the primary driver of the changes in N resorption efficiency of the three dominant grass species. For *L. chinensis*, the P concentration in green and senesced leaves increased the P resorption efficiency, whereas the senesced leaf P concentration played an important role in the P resorption efficiency of *C. squarrosa*. Grazing directly drove the change in P resorption efficiency of *S. grandis*. Our results suggest that large variations in nutrient resorption patterns among plant species depend on leaf nutritional status and nutrient-use strategies under overgrazing, and indicate that overgrazing may have indirect effects on plant-mediated nutrient cycling via inducing shifts in the dominance of the three plant species.

## Introduction

Plant growth is limited by nutrient availability and uptake in various ecosystems (Chapin III, 1980). Leaf nutrient resorption is a key process that controls nutrient fluxes from plants to soil and the nutrients available for storage and reuse (Millard & Proe, 1993). When plants reuse these nutrients, it improves their nutrient retention and adaptability to the environment, and reduces their dependence on the nutrient supply from the current environment (Aerts & Chapin, 2000; Lü et al., 2012). An estimated 50% of total leaf N is resorbed into plant seeds during leaf senescence (Yuan & Chen, 2009). The process of nutrient resorption exerts a strong influence on carbon and nutrient cycling (Kozovits et al., 2007). Many studies have reported the effect of climate change factors such as temperature (Yuan & Chen, 2009), nitrogen addition (Lü et al., 2013) and precipitation (Lü et al., 2012) on nutrient resorption in plants. Research has also demonstrated that grazing, one of the prominent forms of land use in the pastoral areas, affects plant growth (Li et al., 2015) and further impacts soil resource availability and plant nutrient status (Wang et al., 2014). Changes in the biogeochemical cycles of grassland ecosystems may have significant implications for plant nutrient resorption (Lü et al., 2013; Ngatia et al., 2015). However, there is a paucity of information on how grazing impacts nutrient resorption in plants and this study sought to fill this knowledge gap.

Moreover, the effect of grazing on plant diversity, community composition, and ecosystem functioning is well documented in the literature (Koerner & Collins, 2014; Liu et al., 2015). Over the past 50 years, varying levels of degradation have been reported on Chinese grasslands due to overgrazing (Wen et al., 2013). Overgrazing inhibits the growth of palatable and nutrient-rich species with high nutritive value and promotes the dominance of nutrient-poor species or inedible pastures. This phenomenon leads to a decline in nutrient cycling and consequently results in grassland degradation (Ritchie, Tilman & Knops, 1998; Bai et al., 2012). Overgrazing potentially alters plant nutrient resorption through different mechanisms (Lü et al., 2015; Millett & Edmondson, 2015; Ngatia et al., 2015). Firstly, leaf nutrient resorption is impacted by livestock grazing (Millett et al., 2005) depending on the plant’s growth stage (Yasumura et al., 2005), thereby changing the plant community structure and productivity, soil microbial communities, as well as the physical and chemical properties of the soil (Li et al., 2008; He et al., 2011). However, the outcome and magnitude of the grazing effect on plant nutrient resorption processes remain unclear (Ngatia et al., 2015). Secondly, the preference of livestock for different forage species, trampling of the soil, and the return of excreta to the soil alters plant N and P concentrations (Heyburn et al., 2017), which invariably influence plant nutrient resorption. Thirdly, grazing influence nutrient resorption by altering plant phenology, leaf chemistry, and the timing of litterfall which determines the amount of organic matter that is returned back into the soil (Chapman et al., 2006). Moreover, long-term grazing reduces the accumulation of litter, which leads to a reduction in soil water content (Hou et al., 2019). The change in soil water content has an important effect on plant N and P concentrations (Bai et al., 2012) which regulate plant nutrient resorption (Minoletti & Boerner, 1994). Notably, soil water availability has a positive effect on soil N transformation and availability (Wang et al., 2006).

The responses of plant nutrient resorption to overgrazing is dependent on many complex and connected processes, which makes it difficult to predict leaf N and P resorption in the context of land use and management. *L. chinensis* (tall, perennial C_3_ rhizome grass), *S. grandis* (tall, perennial C_3_ bunchgrass), and *C. squarrosa* (short, perennial C_4_ bunchgrass) are three dominant species distributed widely in the typical steppe of the Mongolian plateau. The effects of overgrazing on different species result from complex interactions among forage production, quality and phenology (Ehleringer & Monson, 1993). Previous studies have shown that overgrazing strongly inhibits the growth of *L. chinensis* and *S. grandis* (Liang et al., 2009; Xie & Wittig, 2003), whereas *C. squarrosa* is relatively tolerant to grazing (Liang, Michalk & Millar, 2002). In this study, we investigate how long-term overgrazing affects the plants’ leaf nutrient status and nutrient resorption in three dominant species in the semiarid grasslands of northern China. Specifically, the objectives of this study are to understand: (1) the effects of long-term overgrazing on N and P resorption efficiency and their relationships with plant nutrient concentrations; and (2) the patterns of N and P resorption in three plant species with contrasting overgrazing regulation strategies.

## Materials and Methods

### Study sites and field sampling

The study area was conducted at the Inner Mongolia Grassland Ecosystem Research Station (43°38′N, 116°42′E), located in a typical steppe on the Baiyinxile Ranch, Xilinguole in Inner Mongolia, China. The mean annual temperature is 0.7 °C, and the mean annual precipitation is approximately 350 mm, which occurs mainly in the summer period from June to August. The annual precipitation in 2013 (273.4 mm) and 2014 (256 mm) were lower than the long-term average (278.7 mm). The growing season runs from early April to late September for perennial plant species. The dominant species in the plant community are *Leymus chinensis* (Trin.) Tzvel., *Stipa grandis* P. Smirn., and *Cleistogenes squarrosa* (Trin. ex Ledeb.) Keng, which account for 60–80% of the total biomass in the sward. The sub-dominant species are Keng, *Achnatherum sibiricum* (Linn.) Keng, *Agropyron cristatum* (L.) P. Beauv., *Caragana microphylla* Lam., *Artemisia commutata* Besser, *Carex korshinskyi* Kom., *Kochia prostrata* (L.) Schrad., *Serratula centauroides* L*.*, *Koeleria cristata* (L.) Pers.*, Artemisia frigida* Willd. Sp. Pl. and *Potentilla bifurca* L. This community is representative of one of the most widely distributed grasslands in the Eurasian steppes. The soils are classified as calcic chernozems (IUSS Working Group WRB, 2006).

### Experimental design

We used long-term freely grazed land and a fenced-off land (i.e., grazing exclusion plot) that were established in 1983. At the time of grazing exclusion, the site was considered to be in excellent condition and representative of an undisturbed community. The grazing plot (∼200 ha in area) is located adjacent to the grazing exclusion plot. The plot has been subjected to grazing by ∼600 sheep and goats year-round for more than 30 years at a stocking rate of ∼3 sheep units per hectare. This stocking rate exceeds the local stocking rate of 1.5 sheep units per hectare recommended by the local government for the maintenance of grass-livestock balance.

### Plant sampling and measurement

In this study, we adopted pseudoreplication and space-for-time substitution limitation (Hurlbert, 1984; Walker et al., 2010; Blois et al., 2013; Lü et al., 2014). The sampling area for the grazed and exclusion treatments was 20 × 20 m, established in pairs along transects. There were fifteen replications for each treatment and the plots were allocated 10 m apart along transects. Prior to the commencement of grazing in each grazed plot, three temporary movable exclusion cages (1. 5 × 1.5 m) were set up at each sampling point before the growing seasons in early April 2013 and 2014. Three 1-m^2^ quadrats were established in the three temporary movable exclusion cages for field investigation and sampling. Subsequently, three 1-m^2^ quadrats were used to collect samples for evaluating nutrient resorption efficiency and measure aboveground net primary productivity (ANPP).

During the peak growing period (middle of August 2013 and 2014), representative mature green leaves (the ten visible leaves from the top of the shoot) of three dominant species (*L. chinensis*, *S. grandis* and *C. squarrosa*) were sampled in three 1-m^2^ quadrats. We used the criterion described by Wright & Westoby (2003) to determine leaves ready to abscise. In the middle of October 2013 and 2014, we collected the same number of senesced leaves (ten recently senesced leaves in three 1-m ^2^ quadrats) following the aforementioned procedure. The sampled leaves had no obvious leaf area losses and were transported to the laboratory for analysis. The samples were oven-dried at 70 °C for 48 h and subsequently weighed to obtain sample dry weight. The leaves of each species were bulked per plot and passed through a 40 mm mesh screen using a mechanical mill.

All living vascular plants were clipped (1-m^2^ quadrats) to the ground level for the measurement of ANPP in the current year in each treatment. The collected samples were sorted into species, dried at 70 °C for 48 h in the laboratory and weighed for each quadrat separately. ANPP was calculated as the sum of the aboveground biomass of all vascular plant species based on quadrat.

### Soil sampling and physicochemical properties analysis

We collected soil samples to a depth of 20 cm using an auger (7 cm in diameter and 20 cm long) after the litter layer was removed. Soil organic C (SOC) was determined using the dichromate oxidation method (Nelson & Sommers, 1982). Soil total nitrogen (STN) was measured using micro-Kjeldahl digestion (Nelson & Sommers, 1980). The NH_4_^+^ and NO_3_^−^ concentrations were measured using a continuous-flow auto-analyzer (Alpkem, OI Analytical, USA) after sample extraction by 2 M KCl at a soil: KCl ratio of 1:5 (w:v). Soil available phosphorus (SAP) was determined using the Kelowna method as described by Van Lierop (1988) with a solid to liquid ratio of 1:5. The P concentration in the extracted solution was determined using an Astoria auto-analyzer (Clackamas, OR, Oregon, USA).

### Calculations and statistical analysis

According to Aerts et al. (2007), nitrogen resorption efficiency (NRE) and phosphorus resorption efficiency (PRE) can be calculated based on the total N and P pool in green and senesced leaves. Given that we collected the same number of green and senesced leaves for each species in each plot, nutrient resorption efficiency (RE) was calculated as follows:

NRE = [1 –(Nutrient_senesced_/Nutrient_green_)] ×100%

Where Nutrient_senesced_ and Nutrient_green_ are the N and P pool in the senesced leaves collected in mid-October 2008 and the green leaves sampled in mid-August 2008, respectively. The values obtained for N and P in each species were multiplied by the respective biomass to obtain the species N and P concentrations per unit area (1 × 1 m). Resorption efficiency is the level to which the nutrient concentrations are reduced in the senesced leaves (Killingbeck, 1996; Ratnam et al., 2008). We quantified nutrient resorption efficiency based on the nutrient concentration of the senesced leaves (Killingbeck, 1996), with a lower leaf nutrient concentration indicating a higher nutrient resorption efficiency

### Data analysis

Three-way ANOVA was used to examine the effects of species, treatment, year, and their possible interactions on the green and senesced leaf N contents and N resorption efficiency. We used two-way ANOVA to examine the effects of year and treatment on the soil water content (SWC), SOC, STN, soil total phosphorus (STP), soil inorganic nitrogen (SIN: the sum of NH_4_^+^ and NO_3_^−^ concentrations), SAP, green and senesced leaf N and P contents, and N and P resorption efficiency. One-way ANOVA was used to test for variation in the green and senesced leaf N contents and N resorption efficiency of the three dominant plant species, We used stepwise multiple linear regression to examine the relationships between the soil inorganic nitrogen content and green leaf nitrogen content. A similar regression model was established between soil available phosphorus content and green leaf phosphorus content.

Structural equation models (SEMs) were conducted using IBM SPSS Amos version 21 statistical software (Amos Development Co., Armonk, NY, USA). We hypothesize that the pathways responsible for the effects of grazing on N resorption efficiency are influenced by the green and senesced leaf nitrogen contents and soil inorganic nitrogen content. Similarly, we also hypothesize that the effects of grazing on P resorption efficiency is influenced by the green and senesced leaf nitrogen contents and available phosphorus content. A Chi-square (*χ*^2^) test with the associated probability, the root mean square error of approximation (RMSEA) with the associated probability, and the Comparative Fit Index (CFI) were used to evaluate the fitness of the model. Non-significant *χ*^2^ and RMSEA (*P* > 0.05), and CFI > 0.90 indicate the good fit of SEMs. In addition, the significance of each path in the model depends on the probability level (*P* < 0.05).

## Results

### Responses of the key soil resources and plant biomass

**Figure 1:**
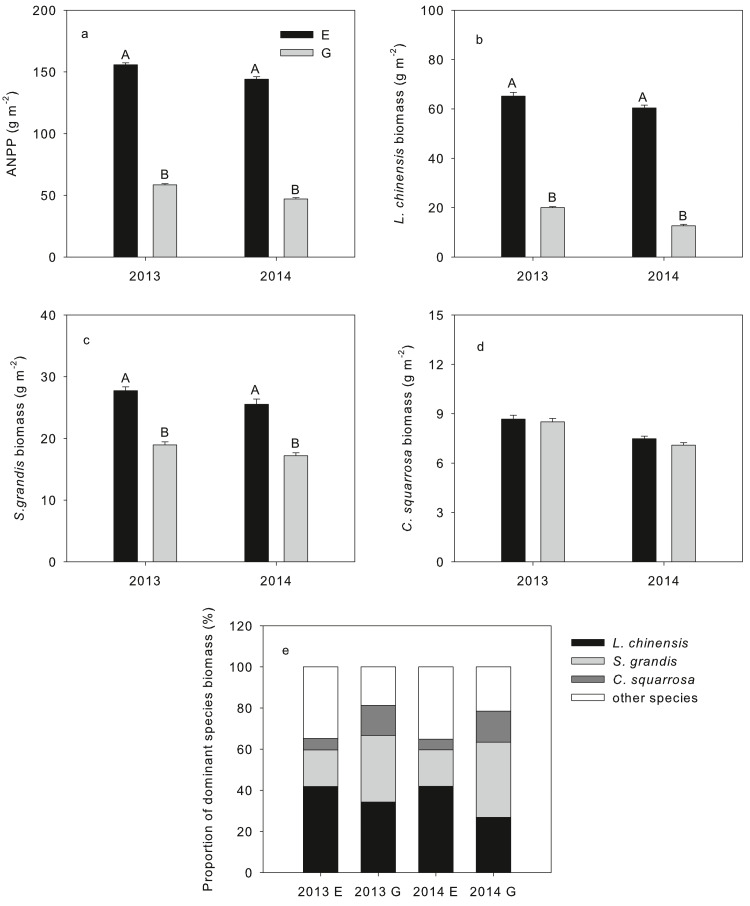
ANPP, the biomass of three dominant species and proportion of dominant species in the community from typical steppe of Inner Mongolia in grazing exclusion and grazing both in 2013 and 2014. Above-ground net primary productivity (ANPP) (A), the biomass of *L. chinensis* (B), *S. grandis* (C) and *C. squarrosa* (D) and proportion of dominant species in the community (E) from typical steppe of Inner Mongolia in grazing exclusion (E) and grazing (G) both in 2013 and 2014. Error bars are SE (*N* = 15). Different letters indicate significant differences (*P* < 0.05) between grazing exclusion and grazing for each species.

Compared with the exclosure, long-term overgrazing significantly decreased the SWC, SOC, STN, and SIN content, whereas the SAP content was significantly enhanced by long-term grazing (*P* < 0.01, Table 1). Some of the key soil resources (SWC, SIN, and SAP) were higher in 2013 than in 2014. The three dominant plant species investigated in this study accounted for >65% of the ANPP in grazed and grazing exclusion plots, respectively (Fig. 1). Long-term grazing significantly decreased the dominant plant species biomass in both years of sampling (*P* < 0.05, Fig. 1). However, *L. chinensis* recorded a higher proportion of biomass in the grazing exclusion plot in both years (41.84% in 2013 and 41.93% in 2014) of sampling, while the biomass of *S. grandis* (36.54%) was higher in the grazed plot in 2014 compared with all other species (Fig. 1E).

**Table 1:** Soil characteristics (0–20 cm) across the 30 years grazing and enclosure in a temperate steppe of northern China.

		SWC	SOC	STN	STP	SIN	SAP
		(%)	(g kg^−1^)	(g kg^−1^)	(g kg^−1^)	(mg kg^−1^)	(mg kg^−1^)
Treatment	Enclosure	9.03^a^	20.49^a^	1.83^a^	0.38	22.42^a^	2.96^b^
	Grazing	8.44^b^	17.40^b^	1.44^b^	0.37	13.48^b^	4.15^a^
Year	2013	9.24^a^	19.37	1.64	0.38	19.55^a^	3.86^a^
	2014	8.24^b^	18.51	1.63	0.37	16.35^b^	3.24^b^
*P* value	Y	<0.0001	0.071	0.735	0.53	<0.0001	<0.0001
	T	<0.0001	<0.0001	<0.0001	0.134	<0.0001	<0.0001
	Y × T	0.194	0.219	0.785	0.971	0.015	0.057

**Notes.**

SWC soil water content SOC soil organic carbon content STN soil total nitrogen content STP soil total phosphorus content SIN soil inorganic nitrogen SAP soil available phosphorus content

a,b Values in the same column with different letters are significantly different (*P* < 0.05).

Y year T treatment

### Responses of leaf nutrient concentration and N resorption efficiency to long-term grazing

The N (18.48 mg g^−1^) and P (1.25 mg g^−1^) concentrations were higher in the grazed plots compared with the grazing exclusion plots (Table 2). Both the green and senesced leaves N concentrations were higher in 2013 than in 2014 (*P* < 0.001), but there was no difference in N resorption efficiency between the years (Table 2). Nitrogen resorption efficiency was higher in the grazed plot than grazing exclusion plots (*P* < 0.001, Table 2). The P resorption efficiency was higher in 2014 than in 2013 (*P* < 0.001, Table 2).

**Table 2:** Results of three-way ANOVAs on the effects of species (*L. chinensis*, *S. grandis* and *C. squarrosa*), treatment (E: grazing exclusion and G: overgrazing), year (2013 and 2014) and their interactions on N and P concentrations in green leaves (Ng and Pg) and senesced leaves (Ns and Ps), N resorption efficiency (NRE) and P resorption efficiency (PRE) in the typical steppe.

		Ng	Pg	Ns	Ps	NRE	PRE
		(mg g^−1^)	(mg g^−1^)	(mg g^−1^)	(mg g^−1^)	(%)	(%)
Species	*L. chinensis*	20.69^a^	1.32^a^	7.79^a^	0.60^b^	69.12^a^	64.71^a^
	*S. grandis*	17.26^b^	1.11^c^	8.02^a^	0.51^c^	64.23^b^	64.87^a^
	*C. squarrosa*	15.75^c^	1.23^b^	6.87^b^	0.73^a^	53.85^c^	48.73^b^
Treatment	E	17.33^b^	1.19^b^	7.93a	0.57^b^	59.62^b^	59.45
	G	18.48^a^	1.25^a^	7.19^b^	0.65^a^	65.19^a^	59.43
Year	2013	18.43^a^	1.24^a^	7.75^a^	0.64^a^	62.71	57.89^b^
	2014	17.37^b^	1.20^b^	7.37^b^	0.58^b^	62.10	60.98^a^
*P* value	S	<0.0001	<0.0001	<0.0001	<0.0001	<0.0001	<0.0001
	T	<0.0001	<0.0001	<0.0001	<0.0001	<0.0001	0.985
	S × G	<0.0001	<0.0001	<0.0001	<0.0001	<0.001	<0.0001
	Y	<0.0001	<0.0001	<0.0001	<0.0001	<0.001	<0.0001
	S × Y	<0.0001	0.049	<0.0001	0.173	0.042	0.341
	T × Y	0.722	0.165	0.041	<0.0001	0.380	<0.001
	S × T × Y	0.042	0.116	<0.0001	0.944	0.528	0.101

**Notes.**

a,b,c Values in the same column with different letters are significantly different (*P* < 0.05).

S plant species T treatment Y year

When we further analyzed the effects of grazing on each species individually, we found that grazing significantly increased the N concentration of *L. chinensis* (21.69 mg g^−1^; *P* < 0.001) and *S. grandis* (18.04 mg g^−1^; *P* < 0.001), but not of *C. squarrosa* (Table 3). Nitrogen resorption efficiency of both *L. chinensis* and *S. grandis* was higher in the grazed plot than grazing exclusion plots (*P* < 0.001, Table 3). In contrast, grazing significantly decreased nitrogen resorption efficiency of *C. squarrosa* (*P* = 0.001, Table 3). Grazing significantly increased the green and senesced leaves P concentrations of the *L. chinensis* (*P* < 0.001, Table 3)*.* Long-term grazing significantly increased the P resorption efficiency of *S. grandis* and decreased that of *C. squarrosa* (*P* < 0.001, Table 3).

**Table 3:** Results of two-way ANOVAs on the effects of treatment (E: grazing exclusion; G: overgrazing), year and their interaction on nitrogen and phosphorus concentration in green leaves (Ng and Pg) and senesced leaves (Ns and Ps), nitrogen resorption efficiency (NRE) and phosphorus resorption efficiency (PRE) for each species in the typical steppe.

		Treatment		Year		*P* value		
		E	G	2013	2014	T	Y	T × Y
*L. chinensis*	Ng	19.69^b^	21.69^a^	21.57^a^	19.81^b^	<0.0001	<0.0001	0.720
	Ns	8.91^a^	6.66^b^	7.78	7.80	<0.0001	0.808	<0.0001
	Pg	1.26^b^	1.39^a^	1.34^a^	1.30^b^	<0.0001	<0.0001	0.502
	Ps	0.55^b^	0.63^a^	0.62^a^	0.58^b^	<0.0001	0.022	0.021
	NRE	63.26^b^	74.99a	69.21	69.04	<0.0001	0.713	0.005
	PRE	63.96	65.47	63.70	65.73	0.828	0.157	0.287
*S. grandis*	Ng	16.48^b^	18.04^a^	17.90^a^	16.62^b^	<0.0001	<0.0001	0.084
	Ns	7.87	8.18	8.11	7.94	0.085	0.360	0.467
	Pg	1.09^b^	1.14a	1.12	1.10	<0.0001	0.064	0.029
	Ps	0.50	0.52	0.54a	0.48^b^	0.102	<0.0001	0.003
	NRE	60.15^b^	68.32^a^	64.64	63.84	<0.0001	0.416	0.764
	PRE	61.55^b^	68.20a	62.66^b^	67.09a	<0.0001	<0.0001	<0.0001
*C. squarrosa*	Ng	15.81	15.69	15.82	15.68	0.356	0.257	0.148
	Ns	6.72^b^	7.02^a^	7.37^a^	6.37^b^	0.046	<0.0001	0.448
	Pg	1.22	1.24	1.25^a^	1.21^b^	0.074	<0.0001	0.387
	Ps	0.66^b^	0.79^a^	0.77^a^	0.69^b^	<0.0001	<0.0001	0.015
	NRE	55.46^a^	52.25^b^	54.28	53.32	0.001	0.358	0.221
	PRE	52.83^a^	44.63^b^	47.32^b^	50.13^a^	<0.0001	0.021	0.016

**Notes.**

a,b Values in the same column with different letters are significantly different (*P* < 0.05).

T treatment Y year

**Figure 2:**
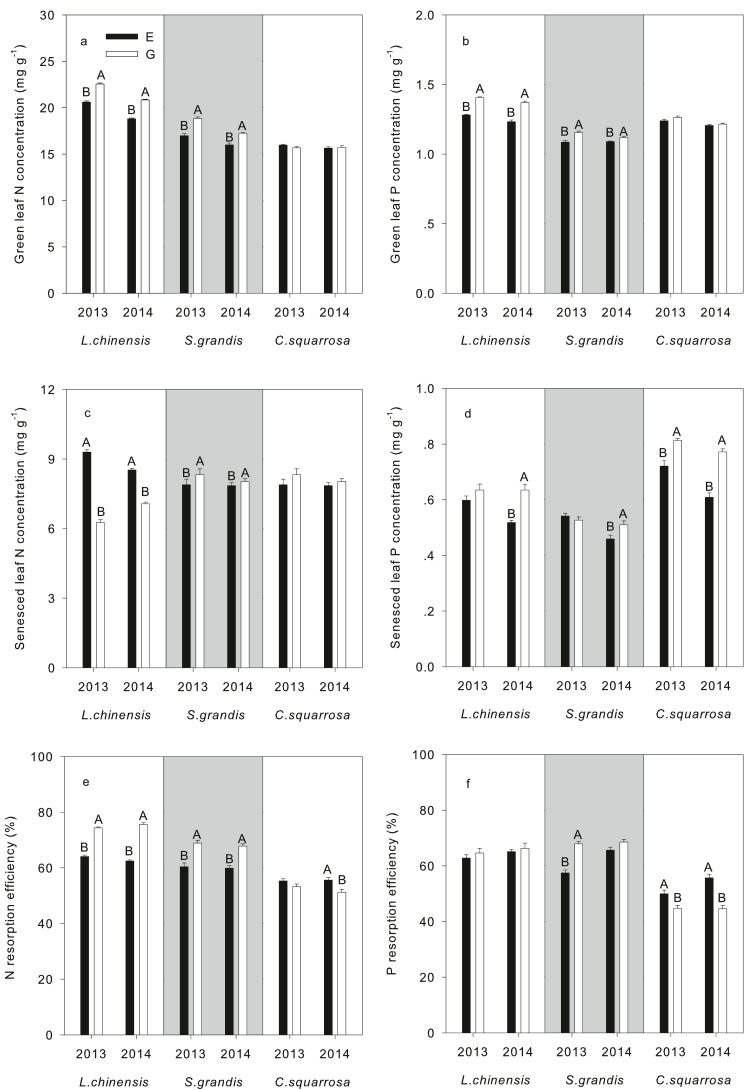
Nitrogen concentrations, nitrogen and phosphorus resorption efficiency of three dominant species from typical steppe of Inner Mongolia in grazing exclusion and overgrazing both in 2013 and 2014. Nitrogen concentrations in green (A, B) and senesced (C, D) leaves and nitrogen and phosphorus resorption efficiency (E, F) of three dominant species from typical steppe of Inner Mongolia in grazing exclusion (E) and overgrazing (G) both in 2013 and 2014. Error bars are SE (*N* = 15). Different letters indicate significant differences (*P* < 0.05) among treatments for each species.

Leaf nutritional traits of the three dominant species responded differently to grazing exclusion (Fig. 2). Green leaf N concentration of *L. chinensis* was the highest among the three dominant species (*P* < 0.05, Fig. 2). The green leaf N and P concentrations of *L. chinensis* and *S. grandis* were higher under long-term grazing than under grazing exclusion both in 2013 and 2014 (*P* < 0.05, Figs. 2A, 2B). Long-term grazing significantly decreased senesced leaf N concentration of *L. chinensis* but increased that of *S. grandis* in 2013 and 2014 (*P* < 0.05, Fig. 2C). In contrast, the senesced P leaf concentration of *C. squarrosa* was enhanced by long-term grazing in both years of sampling (*P* < 0.05, Fig. 2D). There was an increase in the N resorption efficiency of *L. chinensis* and *S. grandis* in the long-term grazing plot in 2013 and 2014, whereas a decrease in N resorption efficiency was recorded for *C. squarrosa* in 2014 (*P* < 0.05, Fig. 2E). In 2013, P resorption efficiency was higher in the grazed plot for *S. grandis* and lower in the same plot for *C. squarrosa* in both years of sampling (*P* < 0.05, Fig. 2F).

### Impact of plant and soil properties on N resorption efficiency under long-term grazing

**Figure 3:**
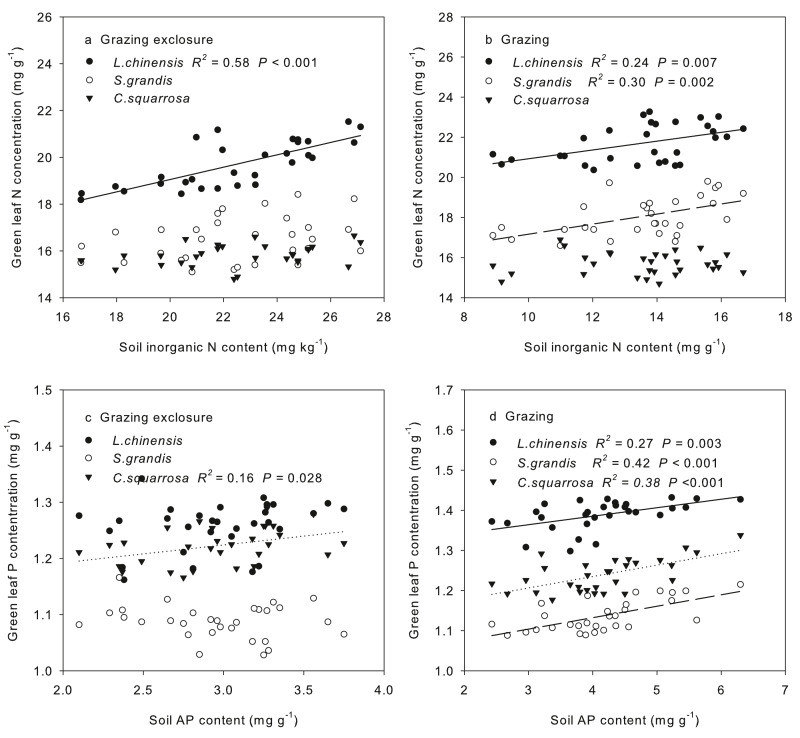
Spatial dependence of green leaf nitrogen concentrations on soil inorganic N content, and spatial dependence of green leaf phosphorus content on soil available phosphorus content. Spatial dependence of green leaf nitrogen concentrations on soil inorganic N content in exclusion (A) and grazing (B), and spatial dependence of green leaf phosphorus content on soil available phosphorus content in grazing exclusion (C) and overgrazing (D) across the 30 plots in two years.

**Figure 4:**
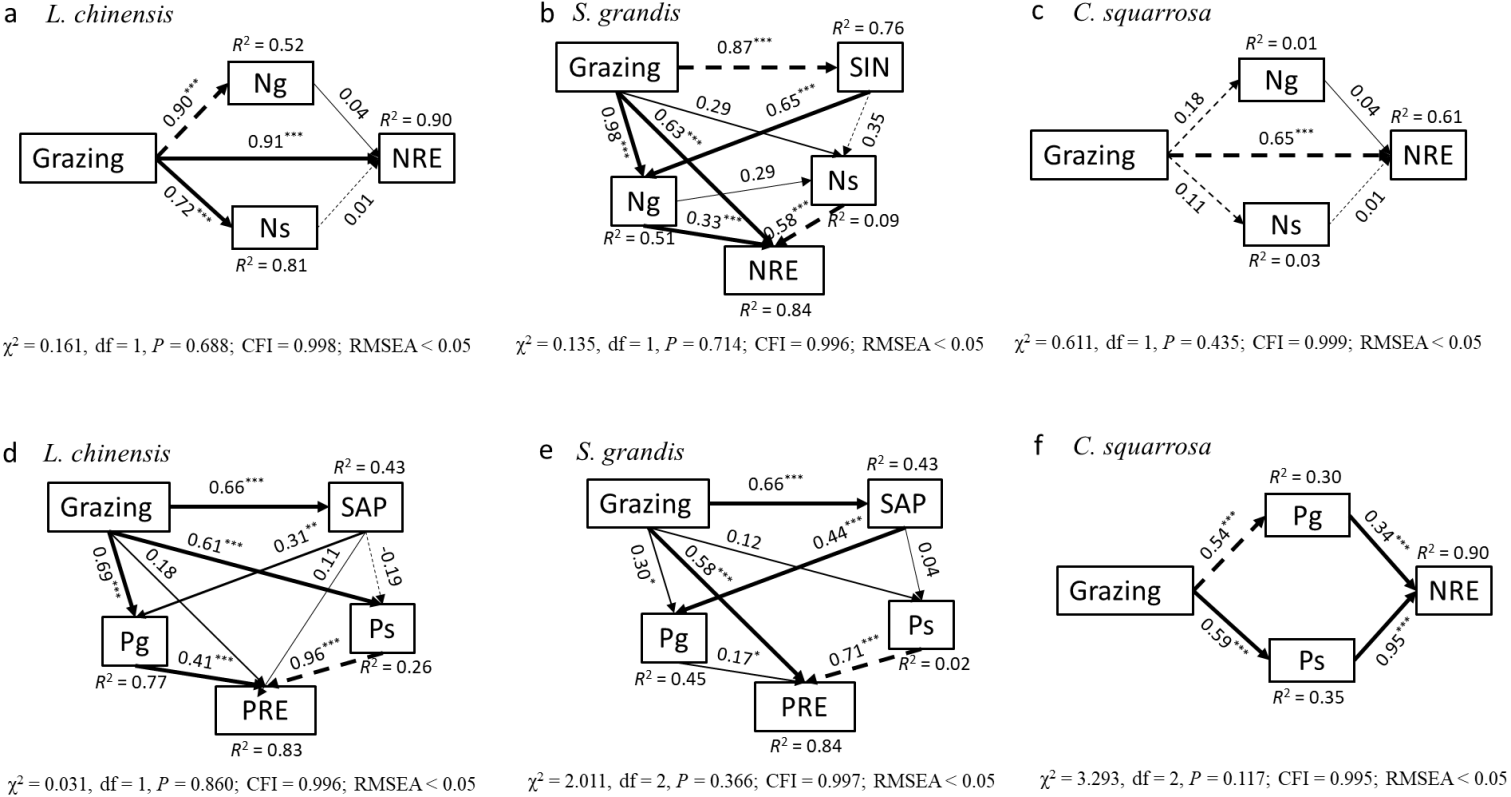
Path analyses on the impacts of and path analyses on the impacts of overgrazing on nitrogen resorption efficiency and phosphorus resorption efficiency of three dominant plant species. Path analyses on the impacts of and path analyses on the impacts of overgrazing on nitrogen resorption efficiency (A, B, C) and phosphorus resorption efficiency (D, E, F) of three dominant plant species (*L. chinensis*; *G. grandis*; *S*. *squarrosa*).****Solid and dashed arrows represent significant (*P* < 0.05, marked *; *P* < 0.01, marked **; *P* < 0.001, marked *** in the figure) and non-significant (*P* > 0.05) paths. Values associated with arrows represent standardized path coefficients. SIN, soil inorganic nitrogen content; Ng, nitrogen concentrations of green leaves; Ns, nitrogen concentrations of senesced leaves; NRE, nitrogen resorption efficiency; SAP, available phosphorus content; Pg, phosphorus concentrations of green leaves; Ps, phosphorus concentrations of senesced leaves.

The green leaf N content was positively correlated to SIN content for *L. chinensis* under grazing exclusion (*R*^2^ = 0.58, *P* < 0.001; Fig. 3A). In the long-term grazing plot, the green leaf N concentration of *L. chinensis* (*R*^2^ = 0.24, *P* = 0.007) and *S. grandis* (*R*^2^ = 0.24, *P* = 0.002) were positively correlated to SIN (Fig. 3B) A positive relationship was found between the leaf P contents of *C. squarrosa* and SAP in the grazing exclusion plot (*R*^2^ = 0.16, *P* = 0.028, Fig. 3C). Moreover, the green leaf P content of all the three dominant species was positively correlated to SAP (*L. chinensis*: *R*^2^ = 0.27, *P* = 0.003; *S. grandis*: *R*^2^ = 0.42, *P* < 0.001; *C. squarrosa*: *R*^2^ = 0.38, *P* < 0.001) under long-term grazing (Fig. 3D). The SEMs explained 90%, 84%, 61%, 83%, 84% and 90% of the variance in N and P resorption efficiency of *L. chinensis*, *S. grandis* and *C. squarrosa*, respectively (Figs. 4A, 4F). Our results showed that grazing has a direct effect on N resorption efficiency of *L. chinensis* and *C. squarrosa*, respectively (Figs. 4A, 4C). The direct and indirect effects of grazing on green leaf nitrogen contents (Ng) and senesced leaf nitrogen contents (Ns) altered the N resorption efficiency of *S. grandis* (Fig. 4B), but P resorption efficiency of the plant was influenced by the direct of grazing on green leaf phosphorus contents (Pg) only *S. grandis* (Fig. 4E). The P resorption efficiency of *L. chinensis* was changed by the indirect effects of grazing on Pg and Ps (Fig. 4D). Similarly, the change in P resorption efficiency of *C. squarrosa* was driven by the indirect effects of grazing on Pg and Ps (Fig. 4F).

## Discussion

### Effects of overgrazing on the leaf nutrient status

The findings from this study reveal that overgrazing significantly increased the N concentration in the green leaves of the dominant species investigated. This corroborates earlier reports (Bai et al., 2012; Ma et al., 2019) that herbivore grazing modifies plant N concentration. Compared with the grazing exclusion plot, the high green leaf N concentration recorded for the dominant species (*L. chinensis*, *S. grandis,* and *C. squarrosa*) in the long-term grazing plot may be attributed to increased synthesis of protein by the ribosome to aid plant growth after successive grazing (Elser et al., 2000; Ma et al., 2019). This suggests a positive response of the species under study to grazing activities by livestock. In addition, the increased green leaf N concentrations under long-term grazing potentially improve the plant species compensatory growth to reduce biomass loss as found by Wang et al. (2004). Moreover, the positive correlation between green leaf N concentration and soil inorganic N content in the grazed plot indicates that the return of livestock excreta (i.e., faeces and urine) could have stimulated root exudation of C-rich substances required for increased N mineralization to enhance green leaf N concentration (Bai et al., 2012). Therefore, our result supports the idea that an increase in leaf N concentration of plants is an important mechanism of responding to grazing in grassland ecosystems (Elser et al., 2010; Peñuelas et al., 2013). In the grazing exclusion plot, only *C. squarrosa* recorded a weak positive relationship between its green leaf P content and SAP. Conversely, the green leaf P content of all the dominant species are positively related to SAP in the long-term grazing plot. Interestingly, SAP is higher in the long-term grazing than the grazing exclusion plot. This implies that the decomposition of faeces and urine in the long-term grazing plot enhanced the faster release of P into the soil (Ma et al., 2019), which makes more P available through microbial mineralization for plants uptake (Chen et al., 2004). Therefore, grazing can increase green leaf P concentration indirectly.

### Effects of overgrazing on leaf nutrient resorption

Although the return of plant foliage into the soil as organic matter potentially increases the N and P input into the ecosystem, the low nutrient resorption efficiency of *C. squarrosa* in the long-term grazing plot may be related to a faster rate of nutrient cycling to replenish plant resources lost to grazing. The reduction in SWC, SOC, and STN recorded in our study is consistent with the report by Millett & Edmondson (2015), and this further strengthens the observed grazing-induced changes in nutrient resorption efficiency of the plant species. Millett et al. (2005) showed that low levels of simulated herbivore grazing had no impact on the N resorption efficiency of *Betula pubescens*, but Silla & Escudero (2003) found that herbivory (light grazing) reduced the green leaf N concentration of Mediterranean *Quercus* species with consequent effect on leaf N resorption efficiency. In this study, we found that more than 50% of N is reabsorbed during leaf senescence, suggesting that nutrient resorption is one of the key mechanisms used by plants for nutrient conservation under grazing. Our result indicates that leaf N resorption efficiency is influenced by grazing and different among the dominant species.

With respect to plant nutrient resorption efficiency, our results suggest that the divergent response of leaf N and P resorption efficiency pattern to overgrazing between the three species most likely resulted from the differences in biological features and resource-use strategies. Our results indicate that *L. chinensis*, *S. grandis* and *C. squarrosa* exhibit a different pattern of N and P resorption efficiency under long-term grazing and grazing exclusion. We surmise that species with high leaf N and P concentrations generally have a relatively fast metabolism because of their high growth rates (Elser et al., 2003). Compared with species with low nutrient concentrations, these species with high leaf N and P concentrations need to obtain more resources (Tilman, 1982). Our findings also suggest that *L. chinensis* relies more on plant internal nutrient cycling in overgrazing and more on soil nutrient resources that are still stored in the grassland. Therefore, *L. chinensis* shows high N resorption efficiency for under overgrazing. Compared with *L. chinensis* and *S. grandis*, it takes an additional 6 weeks for *C. squarrosa* to develop after grazing, particularly when the temperatures are more favorable for their growth (Liang, Michalk & Millar, 2002). *C. squarrosa* is more resistant to grazing than *L. chinensis* and *S. grandis* based on avoidance and tolerance traits (Zheng et al., 2011). It is noteworthy, however, that compared with C _4_grasses (e.g., *C. squarrosa*), C_3_ grasses (e.g., *L. chinensis* and *S. grandis*) generally maintain green leaves longer in regions with cold winters and may stay green in cold regions (Chamaillé-Jammes & Bond, 2010; Fanselow et al., 2011). More importantly, the early onset of leaf senescence for *C. squarrosa* in the region of study (i.e., short plant growing period from early June to the end of September) results in nutrient sequestration in dead leaves. The dead leaves of *C. squarrosa* are easily dispersed by wind, leading to a potential nutrient loss in the soil. As a result, grazing decreased leaf N and P resorption efficiency.

In addition, the preference of livestock for different forage species within the grazing environment is related to the observed changes in the nutrient resorption efficiency in the long-term grazing treatment. *L. chinensis* is highly relished by livestock when at the same stage of growth as other grassland species, due to its higher nitrogen concentration and low C/N ratio. Continuous grazing across the seasons of the year decreased the biomass of *L. chinensis* in both years of sampling. Further, soil cannot provide sufficient nutrients to plants requiring a large amount of N for growth. This may have contributed to the increased N resorption efficiency recorded for *L. chinensis* under long-term grazing. This finding agrees with the results of Killingbeck (1996) that plants with high nutrient resorption efficiency tend to have a lower dependence on the soil nutrient pool. Our SEMs also show that overgrazing has a direct effect on the leaf N resorption efficiency of *L. chinensis*. *S. grandis* is usually abundant in drier and N-limited conditions (Chen et al., 2005), while herbivores prefer *L. chinensis* to the former grass. This implies that under long-term grazing condition, *S. grandis* has a better advantage to efficiently utilize nutrients in a nutrient-limited soil, and SIN content plays a vital role in driving the green leaf N concentration of *S. grandis*. The SEMs also showed that green and senesced leaf N concentration play an important role in driving N resorption efficiency under long-term grazing.

Our results show that grazing had strong direct effects on P resorption efficiency of *S. grandis*, but not on P resorption efficiency of *L. chinensis*. *L. chinensis* has a high degree of stoichiometric N:P homeostasis (grazing 15.60 vs grazing exclusion 15.63) compared with *S. grandis* (grazing 15.12 vs grazing exclusion 15.82) in the green leaves (Table 3), which may in part be supported by previous studies in semiarid steppe (Yu et al., 2010; Mariotte et al., 2017). In addition, phosphorus may not be a limiting factor under overgrazing. The increase in soil P concentration could actually result in increased P availability. The consistent change in leaf P concentration of *L. chinensis* in the green leaf and the senesced leaf may result in no change in phosphorus recovery efficiency. The enhanced P resorption efficiency by *S. grandis* accounts for the increased plant-available P in the soils under long-term grazing. This is corroborated by the positive linear relationship between SAP content and the green leaf P concentration of *S. grandis.* SEM also showed that the indirect effect of grazing (via changes in green leaf P concentration of *S. grandis*) is a major driver that changes P resorption efficiency of *S. grandis*. The high green leaf P concentration and the inconsistent change in senesced leaf P concentration of *S. grandis* under long-term grazing may have resulted in its increased P resorption efficiency. Our SEM result suggests that senesced leaf P concentration is the predominant factor that determines P resorption efficiency of *C. squarrosa*. Previous studies (e.g., Osborne, 2008) have shown that C_3_ and C_4_ plant species differ in their sensitivity to frost, where C_4_ plant species had higher green leaf mortality than C_3_ plants after a frost event. In this study, leaf senescence sets in earlier in C_4_ plant species (*C. squarrosa*, late September) than in C_3_ plant species (*L. chinensis* and *S. grandis*, mid-October), which leads to the incomplete P resorption of *C. squarrosa.* Consequently, the nutrient is retained in the plant leaves, and as such, long-term grazing decreased the leaf P resorption efficiency of *C. squarrosa*.

## Conclusions

This study evaluates how long-term overgrazing affects plants’ leaf nutrient status and resorption in three dominant species of northern China. We found that overgrazing increases green leaf nutrient concentrations and enhances ecosystem nutrient cycling in the ecosystem through increasing senesced leaf nutrient concentrations. Overgrazing increased leaf N concentration and N resorption in *L. chinensis* and *S. grandis*, but overgrazing only had strong effects on P resorption efficiency in *S. grandis*. In contrast, overgrazing reduced N and P resorption efficiency of *C. squarrosa*. Our results provide a better understanding of plant internal nutrient retranslocation in response to grassland management. Our studies suggest that the responses of the three dominant plant species nutrient resorption efficiency to overgrazing appear to be species dependent and associated with species differences in physiological characteristics and adaptive strategies.

##  Supplemental Information

10.7717/peerj.9915/supp-1 Supplemental Information 1 Plant species and community biomass raw data

10.7717/peerj.9915/supp-2 Supplemental Information 2 Soil nutrient raw data

10.7717/peerj.9915/supp-3 Supplemental Information 3 Plant species leaf N and P concentrations in green and senesced periods

10.7717/peerj.9915/supp-4 Supplemental Information 4 Nutrient resorption efficiency raw data

## Additional Information and Declarations

### Field Study Permissions

The following information was supplied relating to field study approvals (i.e., approving body and any reference numbers): The study area was conducted at the Inner Mongolia Grassland Ecosystem Research Station (43°38′N, 116°42′E)

### Data Availability

The following information was supplied regarding data availability: The raw measurements available in the Supplemental Files.

## References

[ref-1] Aerts R, Chapin FS (2000). The mineral nutrition of wild plants revisited: a re-evaluation of processes and patterns. Advances in Ecological Research.

[ref-2] Aerts R, Cornelissen JHC, Van Logtestijn RSP, Callaghan TV (2007). Climate change has only a minor impact on nutrient resorption parameters in a high-latitude peatland. Oecologia.

[ref-3] Bai Y, Wu J, Clark CM, Pan Q, Zhang L, Chen S, Wang Q, Han X (2012). Grazing alters ecosystem functioning and C:N:P stoichiometry of grasslands along a regional precipitation gradient. Journal of Applied Ecology.

[ref-4] Blois JL, Williams JW, Fitzpatrick MC, Jackson ST, Ferrier S (2013). Space can substitute for time in predicting climate change effects on biodiversity. Proceedings of the National Academy of Sciences of the United States of America.

[ref-5] Chamaillé-Jammes S, Bond WJ (2010). Will global change improve grazing quality of grassland? A call for a deeper understanding of the effects of shifts from C4 to C3 grasses for large herbivores. Oikos.

[ref-6] Chapin III FS (1980). The mineral nutrition of wild plants. Annual Review of Ecology and Systematics.

[ref-7] Chapman SK, Langley JA, Hart SC, Koch GW (2006). Plants actively control nitrogen cycling: uncorking the microbial bottleneck. New Phytologist.

[ref-8] Chen SP, Bai YF, Zhang LX, Han XG (2005). Comparing physiological responses of two dominant grass species to nitrogen addition in Xilin River Basin of China. Environmental and Experimental Botany.

[ref-9] Chen CR, Condron LM, Davis MR, Sherlock RR (2004). Effects of plant species on microbial biomass phosphorus and phosphatase activity in a range of grassland soils. Biology and Fertility of Soils.

[ref-10] Ehleringer JR, Monson RK (1993). Evolutionary and ecological aspects of photosynthetic pathway variation. Annual Review of Ecology and Systematics.

[ref-11] Elser JJ, Acharya K, Kyle M, Cotner J, Makino W, Markow T, Watts T, Hobbie S, Fagan W, Schade J, Hood J, Sterner RW (2003). Growth ratestoichiometry couplings in diverse biota. Ecology Letters.

[ref-12] Elser J, Fagan WF, Kerkhoff AJ, Swenson NG, Enquist BJ (2010). Biological stoichiometry of plant production: metabolism, scaling and ecological response to global change. New Phytologist.

[ref-13] Elser JJ, Sterner RW, Gorokhova E, Fagan WF, Markow TA, Cotner JB, Harrison JF, Hobbie SE, Odell GM, Weider LW (2000). Biological stoichiometry from genes to ecosystems. Ecology Letters.

[ref-14] Fanselow N, Schönbach P, Gong X, Lin S, Taube F, Loges F, Pan Q, Dittert K (2011). Short-term regrowth responses of four steppe grassland species to grazing intensity, water and nitrogen in Inner Mongolia. Plant and Soil.

[ref-15] He NP, Zhang YH, Yu Q, Chen QS, Pan QM, Zhang GM, Han XG (2011). Grazing intensity impacts soil carbon and nitrogen storage of continental steppe. Ecosphere.

[ref-16] Heyburn J, McKenzie P, Crawley MJ, Fornara DA (2017). Effects of grassland management on plant C:N:P stoichiometry: implications for soil element cycling and storage. Ecosphere.

[ref-17] Hou D, He W, Liu C, Qiao X, Guo K (2019). Litter accumulation alters the abiotic environment and drives community successional changes in two fenced grasslands in Inner Mongolia. Ecology and Evolution.

[ref-18] Hurlbert SH (1984). Hurlbert-1984-pseudoreplication. Ecological Monographs.

[ref-19] Killingbeck KT (1996). Nutrients in senesced leaves: keys to the search for potential resorption and resorption proficiency. Ecology.

[ref-20] Koerner SE, Collins SL (2014). Interactive effects of grazing, drought, and fire on grassland plant communities in North America and South Africa. Ecology.

[ref-21] Kozovits AR, Bustamante MMC, Garofalo CR, Bucci S, Franco AR, Goldstein G, Meinzer FC (2007). Nutrient resorption and patterns of litter production and decomposition in a Neotropical Savanna. Fuctional Ecology.

[ref-22] Li CL, Hao XY, Zhao ML, Han XG, Willims WD (2008). Influence of historic sheep grazing on vegetation and soil properties of a Desert Steppe in Inner Mongolia. Agriculture, Ecosystems & Envrionment.

[ref-23] Li X, Liu Z, Wang Z, Wu X, Li X, Hu J (2015). Pathways of *Leymus chinensis* individual aboveground biomass decline in natural semiarid grassland induced by overgrazing: a study at the plant functional trait scale. PLOS ONE.

[ref-24] Liang Y, Han GD, Zhou H, Zhao ML, Snyman HA, Shan D, Havstad KA (2009). Grazing intensity on vegetation dynamics of a typical steppe in northeast Inner Mongolia. Rangeland Ecol Manage.

[ref-25] Liang C, Michalk DL, Millar GD (2002). The ecology and growth patterns of *Cleistogenes* species in degraded grasslands of eastern Inner Mongolia, China. Journal of Applied Ecology.

[ref-26] Liu N, Kan H, Yang G, Zhang Y (2015). Changes in plant, soil, and microbes in a typical steppe from simulated grazing: explaining potential change in soil C. Ecological Monographs.

[ref-27] Lü XT, Dijkstra FA, Kong DL, Wang ZW, Han XG (2014). Plant nitrogen uptake drives responses of productivity to nitrogen and water addition in a grassland. Scientific Reports.

[ref-28] Lü XT, Freschet GT, Kazakou E, Wang ZW, Zhou LS, Han XG (2015). Contrasting responses in leaf nutrient-use strategies of two dominant grass species along a 30-yr temperate steppe grazing exclusion chronosequence. Plant and Soil.

[ref-29] Lü XT, Kong DL, Pan QM, Simmons ME, Han XG (2012). Nitrogen and water availability interact to affect leaf stoichiometry in a semi-arid grassland. Oecologia.

[ref-30] Lü XT, Reed S, Yu Q, He N, Wang Z, Han XG (2013). Convergent responses of nitrogen and phosphorus resorption to nitrogen inputs in a semiarid grassland. Global Change Biology.

[ref-31] Ma W, Li J, Jimoh SO, Zhang Y, Guo F, Ding Y, Li X, Hou X (2019). Stoichiometric ratios support plant adaption to grazing moderated by soil nutrients and root enzymes. PeerJ.

[ref-32] Mariotte P, Canarini A, Dijkstra FA, Huenneke L (2017). Stoichiometric N:P flexibility and mycorrhizal symbiosis favour plant resistance against drought. Journal of Ecology.

[ref-33] Millard P, Proe MF (1993). Nitrogen uptake, partitioning and internal cycling in Picea sitchensis (Bong.) Carr. as influenced by nitrogen supply. New Phytologist.

[ref-34] Millett J, Edmondson S (2015). The impact of 36 years of grazing management on soil nitrogen (N) supply rate and Salix repens N status and internal cycling in dune slacks. Plant and Soil.

[ref-35] Millett J, Millard P, Hester AJ, McDonald AJS (2005). Do competition and herbivory alter the internal nitrogen dynamics of birch saplings?. New Phytologist.

[ref-36] Minoletti ML, Boerner REJ (1994). Drought and site fertility effects on foliar nitrogen and phosphorus dynamics and nutrient resorption by the forest understory shrub Viburnum acerifolium L. The American Midland Naturalist.

[ref-37] Nelson D, Sommers LE (1980). Total nitrogen analysis for soil and plant tissues. Journal- Association of Official Analytical Chemists.

[ref-38] Nelson D, Sommers LE, Page AL (1982). Total carbon, organic carbon and organic matter. Methods of soil analysis. Part 2: chemical and microbiological properties.

[ref-39] Ngatia LW, Turne BL, Njoka JT, Young TP, Reddy KR (2015). The effects of herbivory and nutrients on plant biomass and carbon storage in Vertisols of an East African savanna. Agriculture, Ecosystem and Environment.

[ref-40] Osborne CP (2008). Atmosphere, ecology and evolution: what drove the Miocene expansion of C4 grasslands?. Journal of Ecology.

[ref-41] Peñuelas J, Poulter B, Sardans J, Clais P, Van der Velde M, Bopp L, Boucher OR, Godderis Y, Hinsinger P, Llusia J, Nardin E, Vicca S, Obersteiner M, Janssens IA (2013). Human-induced nitrogen–phosphorus imbalances alter natural and managed ecosystems across the globe. Nature Communications.

[ref-42] Ratnam J, Sankaran M, Hanan NP, Gran RC, Zambatis N (2008). Nutrient resorption patterns of plant functional groups in a tropical savanna: variation and functional significance. Oecologia.

[ref-43] Ritchie ME, Tilman D, Knops JMH (1998). Herbivore effects on plant and nitrogen dynamics in oak savanna. Ecology.

[ref-44] Silla F, Escudero A (2003). Uptake, demand and internal cycling of nitrogen in saplings of Mediterranean *Quercus* species. Oecologia.

[ref-45] Tilman D (1982). Resource competition and community structure.

[ref-46] Van Lierop W (1988). Effect of EDTA and DTPA on available-P extraction with the Kelowna multiple element extraction. Canadian Journal of Plant Science.

[ref-47] Walker LR, Wardle DA, Bardgett RD, Clarkson BD (2010). The use of chronosequences in studies of ecological succession and soil development. Journal of Ecology.

[ref-48] Wang Z, Li L, Han X, Dong M (2004). DongDo rhizome severing and shoot defoliation affect clonal growth of *Leymus chinensis* at ramet population level?. Acta Oecologica.

[ref-49] Wang Z, Li Y, Hao X, Zhao M, Han G (2014). Responses of plant community coverage to simulated warming and nitrogen addition in a desert steppe in Northern China. Ecological Research.

[ref-50] Wang CH, Wan SQ, Xing XR, Zhang L, Han XG (2006). Temperature and soil moisture interactively affected soil net N mineralization in temperate grassland in Northern China. Soil Biology & Biochemistry.

[ref-51] Wen L, Dong S, Li Y, Li X, Shi J, Wang Y (2013). Effect of degradation intensity on grassland ecosystem services in the Alpine Region of Qinghai-Tibetan Plateau, China. PLOS ONE.

[ref-52] Wright M, Westoby IJ (2003). The leaf size—twig size spectrum and its relationship to other important spectra of variation among species. Oecologia.

[ref-53] Xie YZ, Wittig R (2003). Growth parameters of characteristic species of Stipa steppes in Northern China as indicators of the grazing intensity. Journal of Applied Botany-angewandte Botanik.

[ref-54] Yasumura Y, Onoda Y, Hikosaka K, Hirose T (2005). Nitrogen resorption from leaves under different growth irradiance in three deciduous woody species. Plant Ecology.

[ref-55] Yu Q, Chen Q, Elser JJ, He N, Wu H, Zhang G, Wu J, Bai Y, Han X (2010). Linking stoichiometric homoeostasis with ecosystem structure, functioning and stability. Ecology Letters.

[ref-56] Yuan ZY, Chen YH (2009). Global-scale patterns of nutrient resorption associated with latitude, temperature and precipitation. Global Ecology and Biogeography.

[ref-57] Zheng S, Lan Z, Li W, Shao R, Shan Y, Wan H, Taube F, Bai Y (2011). Differential responses of plant functional trait to grazing between two contrasting dominant C3 and C4 species in a typical steppe of Inner Mongolia, China. Pant and Soil.

